# Treated for psychosis and presenting with prominent speech and language abnormalities: A case report of an adult with a frontal lobe teratoma

**DOI:** 10.4102/sajpsychiatry.v22i1.924

**Published:** 2016-08-31

**Authors:** Gian Lippi, Elna Naude

**Affiliations:** 1 Department of Psychiatry, University of Pretoria, South Africa

## Abstract

We report on a rare case of an adult presenting with psychotic symptoms, including prominent thought form disorder and an aphasia. Further investigation revealed the presence of a mature teratoma in the left frontal lobe, which could have played a pathophysiological role in the development of both the psychosis and the language impairment.

## Introduction

Teratomas of the brain most commonly occur during infancy and childhood, but are still rare and make up only 0.5% of all intracranial tumours.^1,2^ They usually originate near the midline or from the pineal region.^1,3,4^ Other sites include the basal ganglia, suprasellar region and the pituitary fossa.^1,5^ Most sites are therefore supratentorial, more so in infants than in older children,^3^ but rare instances have been described of teratomas in the cerebellar vermis, fourth ventricle and the medulla oblongata.^1,5^ Teratomas contain structures derived from all three germ cell layers and can be mature and benign or be immature and associated with different malignant forms.^3,4,5,6^ They are well defined, often partially cystic and contain multiple elements, such as hair, bone and cartilage.^4,6^ The tumours may produce plasma or cerebrospinal fluid markers such as alpha 1-fetoprotein and human chorionic gonadotropin.^3^

Presenting symptoms depend a lot on tumour location. Disrupted normal function can therefore be focal or more widespread. The tendency of mature, unruptured teratomas to produce relatively mild symptoms is thought to be secondary to their slow growth and propensity to grow into the subarachnoid spaces. They can present initially with minimal neuropsychiatric symptoms, often without neurological signs.^7^ An association has been shown between frontal lobe teratomas and depression, but other tumour-related neuropsychiatric symptoms such as anxiety, apathy, obsessions, compulsions, paranoia, olfactory delusions and auditory and visual hallucinations have been described as a result of ruptured mature teratomas, which were not necessarily located in the frontal lobe.^7,8^ Violence, suicidal tendencies and incoherent speech have also been documented.^8^

With teratomas most frequently found in infancy and childhood,^1,2^ and the 1-year survival rate among infants with intracranial teratomas being 7.2%,^4^ finding a patient where the diagnosis is made after the age of 20 is unique.^1^

## Case report

Permission to report on this case was granted by the Research Ethics Committee of the Faculty of Health Sciences of the University of Pretoria.

A 26-year-old man was arrested for attempted burglary and because of what was described as abnormal behaviour while in custody was sent for psychiatric observation in terms of Sections 77, 78 and 79 of the *Criminal Procedure Act*, No. 51 of 1977, as amended.^9^ The observation was done at Weskoppies Hospital, a tertiary psychiatric hospital in Pretoria, South Africa. Following the observation period, he was diagnosed with schizophrenia, the charges were dropped and he was referred back to Weskoppies Hospital for treatment as an Involuntary Mental Health Care User under the *Mental Health Care Act*, No. 17 of 2002.^10^

Because of the patient’s severe psychotic state, it was not possible to obtain a reliable background history from him. No collateral history was available because no relative had accompanied him at the time of admission and the police could only supply information related to his arrest.

The patient presented as being unkempt and poorly groomed. He moved with ease and showed no signs of agitation, aggression or psychomotor abnormalities, but was uncooperative. During the interview, it was clear that he was psychotic. His speech was extremely disorganised but with a normal tone, melody and rhythm. He suffered from a severe thought form disorder, presenting mostly with loosening of associations, derailment and neologisms. He would usually give inappropriate answers to questions, but when he did start to answer appropriately, he became tangential almost immediately. He presented with partial hearing impairment. Because of the presence of persecutory, grandiose, referential and bizarre delusions, it was clear that he also had a thought content disturbance. Perceptual disturbances were present in the form of auditory hallucinations. Affect was deemed to be appropriate, but it was difficult to assess the patient’s mood because of his psychotic state. He was poorly oriented, portrayed poor judgement and had no insight into his psychiatric problem. The patient was admitted with a working diagnosis of schizophrenia with the possibility of his psychotic symptoms being substance induced.

During the course of his stay in hospital, his psychotic symptoms did not improve significantly despite optimal treatment with two different first-generation antipsychotics. His auditory hallucinations did disappear, but the delusions and disorganised speech remained. Shortly after admission, his speech slowly decreased until he became mute, but returned with the introduction of a higher antipsychotic dose.

Even though he was clearly thought disordered, it started to become clear that he might also have an aphasia. His speech was fluent and had a normal rate and melody, but he presented with poverty of speech content and paraphasic errors (including neologisms). Yet at times his speech would also be agrammatical. His language comprehension and ability to repeat phrases were intact.

Around the time when the evaluation of his aphasia was taking place, it was reported that he had suffered several tonic-clonic seizures during the course of his admission. He was subsequently sent for an electroencephalogram (EEG). The EEG revealed excessive diffuse low-voltage theta slow-wave activity, medium-voltage delta slow-wave activity in the frontal areas maximal on the left and isolated sharp transients in the left frontal area indicative of localised left frontal dysfunction with possible epileptiform features.

At that time, his general physical examination was normal. Neurological examination revealed increased tone in both legs with brisk knee and ankle reflexes, which seemed to have been missed by the doctor on admission. The plantar response was extensor bilaterally, but power, sensation and proprioception in the legs were intact. Neuropsychiatric examination revealed the presence of multiple frontal lobe functional abnormalities. The rest of the neurological examination, including that of the cranial nerves, was normal.

Because of his clinical presentation, treatment-resistant psychosis and the EEG result, he was sent for a computed tomography scan of his brain.

The scan revealed a large left frontal mass lesion with multiple cystic areas, a high-density centre containing calcium and a low-density area containing fat. A magnetic resonance imaging scan was subsequently done, and the report suggested the presence of a large, left frontal lobe, multicystic inhomogenous tumour with characteristics consistent with a ganglioglioma or pleomorphic xanthoastrocytoma.

The tumour was successfully resected. The histology report revealed an irregular, firm tumour 3 *×* 2 *×* 1.5 cm in size. It was cystic with a fibrous wall and contents consisting mostly of blood – although bone fragments and fat were also identified. A diagnosis of a mature teratoma was made.

During the weeks that followed his surgery, there was very little change in his condition – he was still aphasic, thought disordered and delusional, although his seizures were well controlled on anticonvulsant medication. It was during this time that the social worker was finally able to locate his family. They travelled to Pretoria to pick him up. An interview with the family revealed that they had lost touch with him several years before and that he had been presumed dead. They also revealed that the problems started during his final year of high school when he started getting seizures and became hard of hearing.

The patient was discharged into the care of his family with a referral letter to follow-up at a healthcare facility in their province of residence. He was therefore lost to follow-up locally.

## Discussion

Taking into account that intracranial teratomas mostly occur in infants and children, the low incidence of the tumour and its associated mortality rate, finding an adult patient with such a tumour in his left frontal lobe is rare.^1^

Even though it later emerged that the initial presenting symptoms of the tumour were seizures and hearing loss, it was as a result of neuropsychiatric and not neurological symptoms that the patient presented to our institution. Even though the teratoma was located in the frontal lobe, rather than presenting with depression he presented with less frequently described psychosis and aphasia.

The presence of an aphasia is understandable when the tumour location in the left frontal lobe is taken into account, especially when tumour size and proximity to both the Broca’s and the Wernicke’s speech centres and connecting fibres are considered. Features such as intact fluency of speech with normal rate and melody, poverty of speech content and paraphasic errors (including neologisms) pointed towards a fluent aphasia such as a Wernicke’s aphasia. Yet at times his speech would also be agrammatical, more reminiscent of a non-fluent aphasia such as a Broca’s aphasia. Evaluations confirmed that language comprehension was intact, which suggested that the aphasia was expressive rather than receptive in nature. His ability to repeat phrases, along with his intact comprehension and objectively fluent speech made a diagnosis of a conduction aphasia the most likely, indicating the possibility of arcuate fasciculus pathology.^11^

This case highlights the diagnostic difficulties clinicians are faced with when confronted by a patient with incoherent speech. Distinguishing between the disorganised speech of thought form disorder and an aphasia is difficult enough when only one of the two is present, but correctly identifying and diagnosing both when both are present can be extremely complex. Identifying an aphasia in the presence of established thought form disorder was further complicated in this case by the patient’s speech and language skills having to be evaluated in English, which is not his mother tongue, even though he seemingly had a good grasp of the language. There were staff members who could speak his home language and translate and interpret, but because the thought form disorder and aphasia were also present when he spoke his home language, it was not more helpful communicating through translation because his English language skills were adequate, making it easier to pick up speech and language pathology through direct communication than through translation. Identifying often subtle and hidden symptoms of aphasia is already very challenging, and having to do so in the presence of a language barrier in a patient communicating in a language in which he is not the most proficient is particularly tricky.

The absence of initial collateral information about illness symptoms and progression complicated correct initial diagnosis. This case illustrates the importance of a proper organic workup in a patient presenting with psychosis for the first time, and indeed in a patient presenting with aphasia. Such a workup should always include neuroimaging when available.

This case also reminds us of the importance of reviewing the diagnosis, optimising treatment, excluding organic pathology, illiciting substance use and poor adherence to prescribed medication before considering a psychosis to be treatment resistant.

That being said, even after the surgical removal of the tumour, the patient still presented with treatment-resistant psychotic symptoms. The question remains as to whether the tumour played a pathophysiological role in the development of the psychotic symptoms or whether it was a comorbid finding in a patient with an underlying psychotic disorder. The persistence of symptoms post-surgery does suggest that the tumour had caused neuronal damage that could account for both the continued aphasia and the psychotic symptoms.

## References

[1] Tsuzuki N, Kato H, Ishihara S, Miyazawa T, Nawashuro H, Shima K Malignant teratoma of the medulla oblongata in an adult male. Acta Neurochir (Wien). 2001;143:1303–1304. 10.1007/PL00010098 11810399

[2] Canan A, Gülsevin T, Nejat A, et al Neonatal intracranial teratoma. Brain Dev. 2000;22:340–342. 10.1016/S0387-7604(00)00128-5 10891643

[3] Rosario E, Cohen ML, Cohen AR, Nieder ML Pathological case of the month. Arch Pediatr Adolesc Med. 1999;153:649–650. 10.1001/archpedi.153.6.649 10357310

[4] Bavdek M, Altinörs N, Caner H, et al Giant posterior fossa teratoma. Childs Nerv Syst. 1999;15:359–361. 10.1007/s003810050413 10461789

[5] Sawamura Y, Kato T, Ikeda J, Murata J, Tada M, Shirato H Teratomas of the central nervous system: Treatment considerations based on 34 cases. J Neurosurg. 1998;89:728–737. 10.3171/jns.1998.89.5.0728 9817409

[6] Prause JU, Børgesen SE, Carstensen H, et al Cranio-orbital teratoma. Acta Opthalmol Scand. 1996;74(suppl 219):S53–S56. 10.1111/j.1600-0420.1996.tb00388.x 8741121

[7] Detweiler MB, David E, Arif S Ruptured intracranial dermoid cyst presenting with neuropsychiatric symptoms: A case report. S Med J. 2009;102(1):98–100. 10.1097/SMJ.0b013e318188b290 19077771

[8] Takeuchi H, Kubota T, Kabuto M, Izaki K Ruptured suprasellar dermoid cyst presenting olfactory delusion (Eigengeruchs erlebnis). Neurosurgery. 1993;33(1):97–99. 10.1227/00006123-199307000-00015 8355854

[9] *Criminal Procedure Act*, No. 51 of 1977, as amended. Cape Town: Juta, 1977.

[10] *Mental Health Care Act*, No. 17 of 2002. Cape Town: Juta, 2002.

[11] Cummings JL Concise guide to neuropsychiatry and behavioural neurology. 2nd ed. Washington, DC: American Psychiatric Publishing Inc; 2002.

